# Dynamic Recruitment of CDK5RAP2 to Centrosomes Requires Its Association with Dynein

**DOI:** 10.1371/journal.pone.0068523

**Published:** 2013-07-17

**Authors:** Yue Jia, Ka-Wing Fong, Yuk-Kwan Choi, Siu-San See, Robert Z. Qi

**Affiliations:** Division of Life Science and State Key Laboratory of Molecular Neuroscience, Hong Kong University of Science and Technology, Clear Water Bay, Kowloon, Hong Kong, China; Shantou University Medical College, China

## Abstract

CDK5RAP2 is a centrosomal protein known to be involved in the regulation of the γ-tubulin ring complex and thus the organization of microtubule arrays. However, the mechanism by which CDK5RAP2 is itself recruited to centrosomes is poorly understood. We report here that CDK5RAP2 displays highly dynamic attachment to centrosomes in a microtubule-dependent manner. CDK5RAP2 associates with the retrograde transporter dynein-dynactin and contains a sequence motif that binds to dynein light chain 8. Significantly, disruption of cellular dynein-dynactin function reduces the centrosomal level of CDK5RAP2. These results reveal a key role of the dynein-dynactin complex in the dynamic recruitment of CDK5RAP2 to centrosomes.

## Introduction

In animal cells, centrosomes are the principal microtubule-organizing centers that control the temporal and spatial distribution of the microtubule network. To initiate the assembly of microtubule filaments and to anchor a radial array of microtubules, centrosomes require the presence of γ-tubulin, a highly conserved protein that exists in a macromolecular structure called the γ-tubulin ring complex (γTuRC) [1]–[3]. It is therefore of significant interest to identify the molecules that link the γTuRC to centrosomes. CDK5RAP2 is a centrosomal protein whose mutations lead to autosomal recessive primary microcephaly, a disorder caused by defective proliferation and cell-fate determination of neural progenitors during neurogenesis [4]–[6]. We previously showed that CDK5RAP2 associates with the γTuRC and helps attach it to centrosomes [7], [8]. The γTuRC-binding domain in CDK5RAP2 was delineated as a short sequence stretch that is highly conserved in γ-tubulin complex-targeting proteins of lower organisms, including *Drosophila* centrosomin and fission yeast Mto1p and Pcp1p [7]. Furthermore, CDK5RAP2's γTuRC-binding domain stimulates the microtubule-nucleating activity of the γTuRC [8], and a disruption of CDK5RAP2 function results in the disorganization of interphase microtubules and the formation of anastral mitotic spindles [7]. Thus, CDK5RAP2 plays an essential role in the organization of microtubules by centrosomes.

The centrosome's pericentriolar material (PCM) dynamically exchanges its molecular contents with the cytoplasm, and several PCM components, such as the matrix proteins pericentrin and PCM-1, are recruited to centrosomes via microtubule-dependent mechanisms [9]–[11]. These mechanisms require the minus end-directed microtubule motor protein dynein in association with dynactin, a large molecular complex necessary for dynein functions [12],[13]. Cytoplasmic dynein consists of dynein heavy chains (DHCs), dynein intermediate chains (DICs), dynein light intermediate chains (DLICs) and dynein light chains (DLCs). Whereas the DICs interact with most of the other dynein components and with the dynactin complex, the DLICs and DLCs are thought to function in loading cargo onto dynein [14]–[17]. CDK5RAP2, like γ-tubulin and many other centrosomal proteins, is present in a large cytoplasmic pool, but how it is targeted to centrosomes has remained unclear. Here we report that CDK5RAP2 is dynamically recruited to centrosomes in a microtubule-dependent manner and that CDK5RAP2 associates with dynein and depends on the dynein-dynactin complex for its localization at centrosomes.

## Results

### Dynamic attachment of CDK5RAP2 to centrosomes requires microtubules

**Figure 1:**
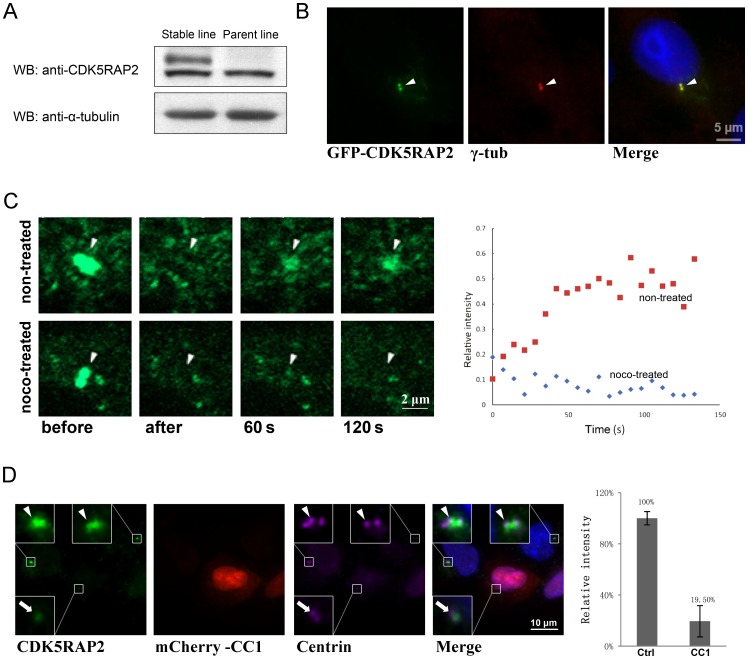
The dynamic recruitment of CDK5RAP2 to centrosomes depends on microtubules and dynein. (A) Immunoblotting of extracts prepared from MDA-MB-231 cells stably expressing GFP-CDK5RAP2. Cells were lysed and extracts were stained with an anti-CDK5RAP2 antibody to detect GFP-CDK5RAP2 and endogenous CDK5RAP2 in either the stable cell line or the parent cell line. Anti-α-tubulin was used as internal control. (B) GFP-CDK5RAP2 is localized on centrosomes. Cells transfected with GFP-CDK5RAP2 were stained with anti-γ-tubulin to label centrosomes. (C) Centrosomal recruitment of CDK5RAP2. FRAP experiments were performed on cells expressing GFP-CDK5RAP2 that were treated with or without nocodazole (noco, nocodazole 10 μM for 1 h) before starting the assays. The intensity of the CDK5RAP2 signal relative to its original level (before bleaching) on centrosomes (arrowheads) is plotted on the right; the intensity recovered to 60% of the original intensity (n = 5). (D) Effect of the disruption of dynein-dynactin on the centrosomal accumulation of CDK5RAP2. The centrosomal level of CDK5RAP2 was determined in cells un-transfected and transfected with mCherry-CC1; relative amounts of centrosomal CDK5RAP2 are shown on the right (n≥30 cells for each case; error bars, S.D. *p*<0.001). Arrows and arrowheads point to centrosomes in CC1-positive and CC1-negative cells. Centrin serves as an indicator of centrosomes. Centrosomes are magnified in insets. Scale bar, 10 μm. Representative results of at least three separate experiments are shown here.

To examine the dynamics of centrosomal CDK5RAP2 by the fluorescence recovery after photobleaching (FRAP), we constructed a stable cell line expressing GFP-CDK5RAP2 at a level similar to that of the endogenous protein (Fig. 1A). GFP-CDK5RAP2 was enriched at centrosomes in these cells (Fig. 1B) and its signal at these sites was then eliminated by photobleaching. The signal, however, reappeared quickly after bleaching, with 50% recovery occurring within ∼1 min and reaching ∼60% of the original fluorescence intensity with prolonged incubation (Fig. 1C). This is in accord with previous observations of CDK5RAP2's dynamic localization at centrosomes [18], [19]. Strikingly, when FRAP was carried out on these cells after treating them with nocodazole to depolymerize microtubules, the GFP-CDK5RAP2 signal failed to recover at centrosomes during the recording period (Fig. 1B, noco-treated). These results demonstrate that intact microtubules are needed for the dynamic recruitment of CDK5RAP2 to centrosomes.

Protein transport along microtubules involves cytoplasmic dynein associated with dynactin, a large complex comprising at least 11 different subunits that include p150^glued^ and dynamitin [20]. Transport of most dynein cargos is blocked when the dynactin complex is disrupted by over-expressing either the coil-coil domain 1 (CC1) of p150^glued^ or the dynamitin protein [21]–[24]. To probe the involvement of dynein-dynactin in the centrosomal targeting of CDK5RAP2, we over expressed CC1 in cells and then examined the centrosomal content of CDK5RAP2. Centrosomes were labeled with an anti-centrin antibody. In the presence of excess CC1, the centrosomal level of CDK5RAP2 was reduced by ∼80% (Fig. 1D), suggesting that dynein-dynactin mediates the transport of CDK5RAP2 towards centrosomes.

### CDK5RAP2 associates with dynein

**Figure 2:**
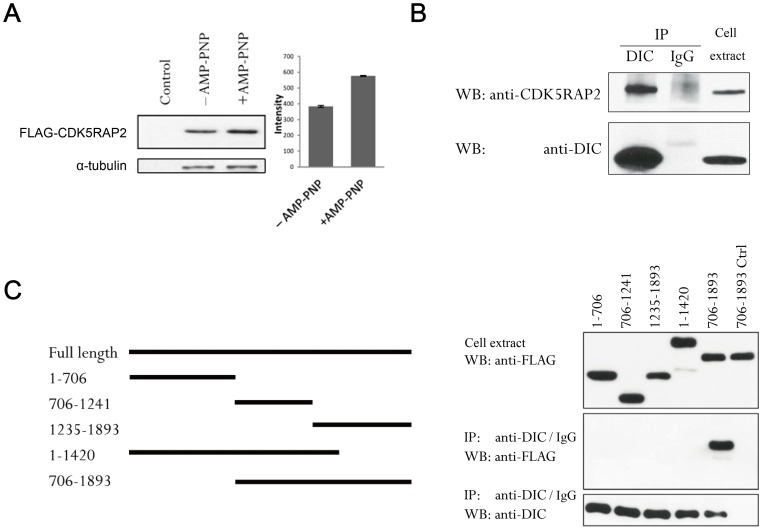
CDK5RAP2 associates with dynein. (A) Extracts of HEK293T cells expressing FLAG-CDK5RAP2 were incubated with taxol-polymerized microtubules in the absence or presence of AMP-PNP; control samples lacked polymerized microtubules. After incubation, microtubules were spun down through a sucrose cushion and the pellets were immunoblotted with an anti-FLAG antibody for CDK5RAP2; α-tubulin in these pellets was quantified by staining with anti-α-tubulin antibody (n = 3, *p*<0.001). (B) DIC was immunoprecipitated from HEK293T extracts using an anti-DIC antibody, normal mouse IgG served as a control. The immunoprecipitates (“IP” here and in other figures) and cell extracts were probed for DIC and CDK5RAP2 by Western blotting (“WB” here and in other figures). (C) Mapping the DIC-binding region in CDK5RAP2. Anti-DIC immunoprecipitation was performed on HEK293T ectopically expressing CDK5RAP2 fragments. Immunoprecipitates and cell extracts were analyzed for DIC and for the CDK5RAP2 fragments with anti-FLAG. A control immunoprecipitation (Ctrl) was performed using normal mouse IgG on lysates of 706–1893-expressing cells.

Previously we showed that CDK5RAP2 associates with microtubules using a microtubule sedimentation assay [7]. When the same sedimentation assay was performed here in the presence of the ATP analog AMP-PNP, which facilitates the binding of molecular motors to microtubules [9], the co-sedimentation of CDK5RAP2 with microtubules was significantly enhanced (Fig. 2A). This indicated the involvement of motor proteins in mediating the association of CDK5RAP2 with microtubules. Thus, to examine the potential interaction of CDK5RAP2 with dynein, DIC, a core component of dynein, was immunoprecipitated. We found that CDK5RAP2 was present in anti-DIC but not control IgG immunoprecipitates (Fig. 2B). Furthermore, DIC co-immunoprecipitated with a FLAG-tagged CDK5RAP2 construct spanning residues 706–1893 but not with construct 1–706, and any truncation of the 706–1893 construct eliminated its association with DIC (Fig. 2C). Therefore, we conclude that a large carboxy-terminal region of CDK5RAP2 mediates its interaction with dynein.

**Figure 3:**
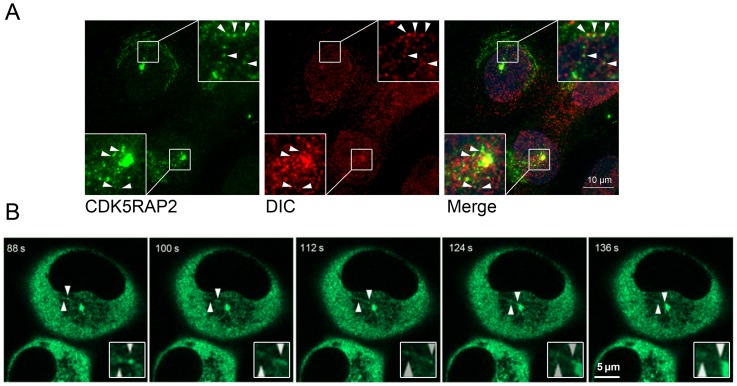
CDK5RAP2 colocalizes with dynein. (A) MDA-MB-231 cells expressing GFP-CDK5RAP2 were stained with anti-DIC and fluorescent secondary antibodies. Arrowheads point to overlapping CDK5RAP2 and DIC signals, which are shown magnified in the insets. Scale bar, 10 μm. (B) Transport of GFP-CDK5RAP2 in living cells. The dynamics of CDK5RAP2 was monitored using MDA-MB-231 cells that stably expressed GFP-CDK5RAP2 at levels similar to that of the endogenous protein. Here a time series is shown for GFP-CDK5RAP2, with time points indicated at the upper left corner. Arrowheads point to a mobile CDK5RAP2-containing particle (again magnified in insets). Images shown here are representatives from at least three independent experiments.

Next, to ask whether CDK5RAP2 and dynein colocalize in cells, immunofluorescence microscopy and time-lapse microscopy were performed. Endogenous CDK5RAP2 was detected in cytoplasmic particles which were also labeled by the anti-DIC antibody (Fig. 3A). The expression of DIC was demonstrated by immunoblotting (Fig. 2B). Time-lapse microscopy carried out on GFP-CDK5RAP2 stably expressed cells showed that the cytoplasmic particles containing GFP-CDK5RAP2 moved along microtubules towards centrosomes (Fig. 3B and Movie S1). The particles traveled at ∼0.5 μm/s, a speed similar to that of motor dynein movement [25], [26]. Collectively, these results point to a dynamic transport of CDK5RAP2 with dynein to centrosomes.

### CDK5RAP2 interacts with dynein light chain 8

**Figure 4:**
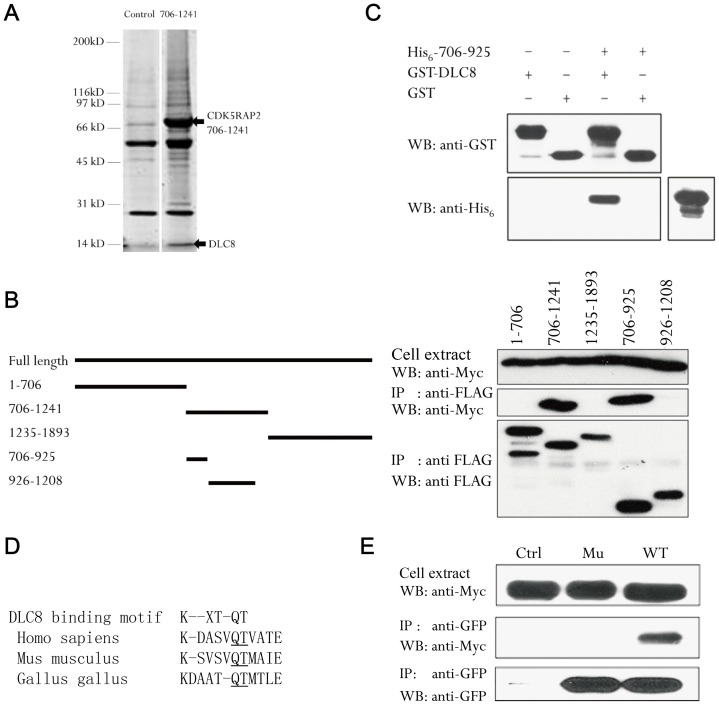
CDK5RAP2 binds to DLC8. (A) Extracts of HEK293T cells transfected with FLAG-CDK5RAP2 (706–1241) or the empty vector were used in immunoprecipitation assays with anti-FLAG. Immunoprecipitated proteins stained on SDS-PAGE gels were digested with trypsin, extracted, and analyzed by mass spectrometry. Arrows show the bands corresponding to DLC8 and the CDK5RAP2 fragment. (B) Mapping the DLC8-binding site in CDK5RAP2. Myc-DLC8 and various CDK5RAP2 fragments (FLAG tagged) were co-transfected into HEK293T cells. After immunoprecipitation with the anti-FLAG antibody, captured samples and cell extracts were probed with anti-FLAG and anti-Myc antibodies. (C) Direct binding of DLC8 to CDK5RAP2. Recombinant CDK5RAP2 (706–925) and DLC8 were subjected to an in vitro binding assay: after pulling-down GST-DLC8 with GSH-beads, anti-His_6_ immunoblotting was performed to detect His_6_-CDK5RAP2 (706–925). (D) Alignment of the DLC8-binding motif in human, mouse and chicken CDK5RAP2. (E) Anti-GFP immunoprecipitation was performed using HEK293T cells co-expressing wild-type or mutant GFP-CDK5RAP2 with Myc-DLC8 and the immunoprecipitates were probed on blots with anti-Myc and anti-GFP antibodies. Ctrl, GFP vector; WT, CDK5RAP2 wild-type; Mu, CDK5RAP2 (Q874A/T875A). Blots shown here are representatives of three separate experiments.

To ascertain whether CDK5RAP2 directly interacts with dynein, we biochemically isolated proteins that bind to CDK5RAP2. In these screens the CDK5RAP2 fragment 706–1241 specifically captured DLC8, which was identified by mass spectrometry (Fig. 4A). To verify this binding we co-transfected cells with CDK5RAP2 fragments and DLC8 for immunoprecipitation. In these assays DLC8 associated with CDK5RAP2 706–1241 and the 706–925 sequence stretch within it, but not with other regions of CDK5RAP2 (Fig. 4B). However, since co-immunoprecipitation of two proteins does not necessarily mean direct interaction between them, we expressed CDK5RAP2 706–925 and DLC8 in bacteria and purified the recombinant proteins for “pull-down” assays: the 706–925 fragment was incubated with GST-DLC8 or GST and then GST was captured using GSH-beads. CDK5RAP2 706-925 was readily detected in pull-downs of GST-DLC8 but not of GST alone (Fig. 4C), indicating the direct binding of 706–925 to DLC8.

DLC8 recognizes and interacts with the motif K/R-X-T-Q-T (where X is any amino acid) [14], and sequence scanning of CDK5RAP2 706–925 revealed such a motif (Fig. 4D). The sequence in this region of human CDK5RAP2 is conserved in mouse and chicken orthologs (Fig. 4D) but is not found in centrosomin from *Drosophila*. Within the DLC8-binding motif, the dipeptide Gln-Thr was previously shown to be involved in mediating the interaction with DLC8 [14]. Thus, we created the double mutation Q874A/T875A in CDK5RAP2 and expressed the mutant and wild-type CDK5RAP2 in cells to test their binding to DLC8 by immunoprecipitation. The double mutation eliminated the DLC8-binding activity of CDK5RAP2 (Fig. 4E), indicating that this short sequence found in 706–925 is critical for CDK5RAP2's interaction with DLC8.

## Discussion

Microtubules help proteins travel to and from centrosomes. Several centrosomal components, such as the matrix proteins pericentrin and PCM-1, are recruited to centrosomes by microtubule-dependent mechanisms, which require the minus end-directed microtubule motor dynein in conjunction with dynactin [9], [21], [26], [27]. Present on centrosomes throughout the cell cycle, CDK5RAP2 plays an important role in the microtubule-organizing function of centrosomes. We have shown here that CDK5RAP2 associates with dynein and that dynein-dependent transport is necessary for the dynamic attachment of CDK5RAP2 to centrosomes. Therefore, this report provides direct evidence for dynein-mediated recruitment of CDK5RAP2 to centrosomes.

CDK5RAP2 localizes dynamically on centrosomes with a turnover rate similar to its homologue centrosomin (Cnn) in *Drosophila* but significantly greater than that of γ-tubulin [19], [28], which suggests that CDK5RAP2 and γ-tubulin are transported separately to centrosomes. The assembly of γ-tubulin on centrosomes appears to involve both microtubule-dependent and microtubule-independent pathways: on the one hand, the γ-tubulin level at centrosomes has been found to depend on intact microtubules and a functional dynein-dynactin complex [21], [26], but on the other hand studies have pointed to γ-tubulin recruitment to centrosomes in the absence of microtubules and presumably by passive diffusion [27], [29]–[33]. It is possible that these two pathways function independently in the recruitment of γ-tubulin, with perhaps the cellular conditions determining which of the two predominates. Our FRAP experiments showed that the recovery of CDK5RAP2 signal at centrosomes relies on the presence of an intact microtubule network (Fig. 1B), which suggests that the microtubule-dependent mechanism plays a principal role in efficiently trafficking CDK5RAP2 to centrosomes; our data, however, do not exclude alternative pathways for the centrosomal recruitment of CDK5RAP2.

In this study we have demonstrated CDK5RAP2's association with the cytoplasmic dynein motor protein and identified a DLC8-binding motif in CDK5RAP2. DLC8, one of the three dimeric light chains of the dynein complex, is a highly conserved protein with diverse functions [34],[35]. It acts as an adaptor between dynein and its cargo proteins [36], but may also have motor-independent functions in activities such as protein folding and dimerization, protein-protein interactions, and nuclear import of proteins [15], [35], [37], [38]. We have found that a large region of CDK5RAP2 encompassing the DLC8-binding domain is required for association with dynein. However, the DLC8-binding domain is insufficient for the interaction of CDK5RAP2 with dynein (Fig. 2C), suggesting that the mechanism of CDK5RAP2's association with dynein is more complex. Dynein associates with dynactin, which regulates dynein functions and also links dynein to various cargos [20], [39]–[41]. We found that the centrosomal content of CDK5RAP2 was significantly reduced following the disruption of the dynein-dynactin complex by over-expression of CC1 of p150^glued^ (Fig. 1C); introduction of excess CC1 produces effects similar to that seen with the over-expression of the dynactin subunit dynamitin [24], [42]. Collectively, our results suggest that CDK5RAP2 associates with the cytoplasmic dynein-dynactin complex for retrograde transport.

In conclusion, CDK5RAP2 plays an important role in the structural organization of the PCM and in the microtubule nucleation that occurs therein [8], [43]–[45]. Our present study showing microtubule-dependent targeting of CDK5RAP2 to centrosomes lends further support to the notion that an intact microtubule network is required for centrosome assembly and functions. The centrosomal levels of CDK5RAP2 may be maintained by its well-controlled recruitment, release, and degradation, and thus the coupling of CDK5RAP2 to dynein-dynactin may directly regulate centrosomal activities.

## Materials and Methods

### DNA cloning

CDK5RAP2 fragments were subcloned into pFLAG-CMV-2 (Sigma-Aldrich) or pET21b (Novagen). Site-directed mutagenesis was performed by a PCR-based method. pGEX4T1-DLC8 was a gift from Dr. Mingjie Zhang (Hong Kong University of Science and Technology, Hong Kong, China). CC1, the p150^glued^ coiled-coil domain 1 (217–548) [21], was amplified by PCR and cloned into the pCI-neo vector (Promega) in fusion with an mCherry tag.

### Antibodies

The polyclonal anti-GFP, anti-GST, and anti-CDK5RAP2 antibodies used in this study were generated in rabbits [7]. The following antibodies were purchased: anti-FLAG (M2 and polyclonal, Sigma-Aldrich), anti-β-tubulin (Sigma-Aldrich), anti-DIC (Millipore), anti-DLC8 (Abgent) and anti-Myc (Santa Cruz).

### Recombinant protein preparation

Recombinant proteins with His_6_ or GST tag were expressed in *Escherichia coli* BL21 (DE3) and then isolated using Ni^2+^-nitrilotriacetic acid resin (Qiagen) or GSH-Sepharose (GE Healthcare), respectively. The isolated proteins were dialyzed in TBS (50 mM Tris-HCl, pH 7.4, 150 mM NaCl, 1 mM EGTA) or PBS (137 mM NaCl, 2.7 mM KCl, 4.3 mM Na_2_HPO_4_, and 1.47 mM KH_2_PO_4_, pH 7.4) containing 10% glycerol before storing at −80°C.

### Cell culture and transfection

HEK293T and MDA-MB-231 cells were cultured on poly-D-lysine coated dishes or on 16-mm coverslips with DMEM or RPMI1640 medium, respectively, supplemented with 10% fetal bovine serum and 1% penicillin/streptomycin. Plasmid transfection was performed using Lipofectamine™ LTX and Plus Reagent or Lipofectamine 2000 (Invitrogen). Stable MDA-MB-231 cell lines expressing GFP-CDK5RAP2 were selected using 400 μg/ml G418 after transfection; individual G418-resistant cells were selected, cultured and tested for expression of GFP-CDK5RAP2 by immunoblotting.

### Microscopic imaging

To perform immunostaining, cells grown on coverslips were fixed with methanol at −20°C or with 4% paraformaldehyde at room temperature, permeabilized with 0.5% Triton X-100 in PBS and then stained with primary antibodies for 2 h at room temperature. After extensive washing with PBS, secondary antibodies coupled with AlexaFluor488 or AlexaFluro594 conjugates (Invitrogen) were used for staining for another 1 h. Nuclear DNA was stained with 1 μM Hoechst 33528 (Sigma-Aldrich). Fluorescent images were acquired with an inverted microscope (Eclipse TE2000, Nikon) or a confocal microscope (LSM510 META, Carl Zeiss Microimaging). To perform FRAP, cells were cultured on 35-mm glass-bottom dishes. Selected regions of cells were bleached at 95% laser power for 20 iterations on the confocal microscope. Two photos were captured before photobleaching and time-lapse photos at 2–4 s intervals were obtained after photobleaching to monitor fluorescence recovery.

### Immunoprecipitation

At 24 h after transfection, cell extracts (1∼2×10^6^ cells for binding assays and 1×10^7^cells for protein identification by mass spectrometry) were prepared in lysis buffer (25 mM Tris-HCl, pH 7.4, 0.5% NP40, 100 mM NaCl, 5 mM MgCl_2_, 5 mM NaF, 1 mM dithiothreitol, Roche Complete Protease Inhibitor Cocktail) and clarified by centrifugation (16,000×*g*, 15 min). Immunoprecipitation was performed for at least 2 h at 4°C with antibodies coupled to Protein A/G Agarose or with ANTI-FLAG^®^ M2 Affinity Gel (Sigma-Aldrich). Immunoprecipitates were resolved by SDS-PAGE for immunoblotting or for total protein staining in gels with Sypro Ruby (Bio-Rad Laboratories). Proteins present in the bands excised from gels were identified by tandem mass spectrometry (LTQ Velos linear ion trap LC-MS system, Thermo Fisher Scientific).

### Protein binding assay

Recombinant proteins (5 μg) with GST or His_6_ tag were mixed in 200 μl binding buffer (25 mM Tris-HCl, pH 7.4, 0.5% NP40, 100 mM NaCl, 5 mM MgCl_2_, 5 mM NaF, 1 mM dithiothreitol, Roche Complete Protease Inhibitor Cocktail, and 1 mg/ml bovine serum albumin). After incubation at 4°C for 1 h, GST proteins were retrieved with GSH-Sepharose to analyze for bound proteins by immunoblotting.

### Microtubule sedimentation assay

Tubulins were purified from porcine brain by two cycles of temperature-dependent assembly/disassembly followed by phosphocellulose chromatography [46]. Cell lysates of CDK5RAP2 were prepared in PEM buffer (80 mM PIPES, pH 6.8, 1 mM MgCl_2_ and 1 mM EGTA) supplemented with 50 mM NaCl, 1% Triton X-100 and the Roche Complete Protease Inhibitor Cocktail; lysates were clarified by spinning them in a refrigerated microcentrifuge at full speed for 15 min. Microtubules were preassembled from purified tubulins (50 µg) in the PEM buffer containing 50 µM taxol (Sigma) and 1 mM GTP and then incubated with the cell lysates in 150 µl PEM buffer containing taxol and GTP for 30 min at room temperature. AMP-PNP was used at 0.5 mM in assays as indicated. Samples were centrifuged through a 20% sucrose cushion at 20,000×*g* for 30 min at 4°C and the resulting pellets were boiled in SDS-PAGE sample buffer and analyzed by immunoblotting.

### Statistical analysis

Statistical analysis was performed with two-tailed Student's *t*-test.

## Supporting Information

Movie S1 GFP-CDK5RAP2 was expressed in MDA-MB-231 cells and observed under a confocal microscope. Live cell images were captured at 12 s-intervals. The arrowheads show the centrosomal-targeting of moving particles; one of these particles first traveled along the microtubules and finally fused with a centrosome.
(ZIP)
